# Natural Strain Variation and Antibody Neutralization of Dengue Serotype 3 Viruses

**DOI:** 10.1371/journal.ppat.1000821

**Published:** 2010-03-19

**Authors:** Wahala M. P. B. Wahala, Eric F. Donaldson, Ruklanthi de Alwis, Mary Ann Accavitti-Loper, Ralph S. Baric, Aravinda M. de Silva

**Affiliations:** 1 Department of Microbiology and Immunology, University of North Carolina School of Medicine, Chapel Hill, North Carolina, United States of America; 2 Department of Epidemiology, Gillings School of Global Health, University of North Carolina, Chapel Hill, North Carolina, United States of America; 3 Department of Medicine, University of Alabama at Birmingham, Birmingham, Alabama, United States of America; Oregon Health & Science University, United States of America

## Abstract

Dengue viruses (DENVs) are emerging, mosquito-borne flaviviruses which cause dengue fever and dengue hemorrhagic fever. The DENV complex consists of 4 serotypes designated DENV1-DENV4. Following natural infection with DENV, individuals develop serotype specific, neutralizing antibody responses. Monoclonal antibodies (MAbs) have been used to map neutralizing epitopes on dengue and other flaviviruses. Most serotype-specific, neutralizing MAbs bind to the lateral ridge of domain III of E protein (EDIII). It has been widely assumed that the EDIII lateral ridge epitope is conserved within each DENV serotype and a good target for vaccines. Using phylogenetic methods, we compared the amino acid sequence of 175 E proteins representing the different genotypes of DENV3 and identified a panel of surface exposed amino acids, including residues in EDIII, that are highly variant across the four DENV3 genotypes. The variable amino acids include six residues at the lateral ridge of EDIII. We used a panel of DENV3 mouse MAbs to assess the functional significance of naturally occurring amino acid variation. From the panel of antibodies, we identified three neutralizing MAbs that bound to EDIII of DENV3. Recombinant proteins and naturally occurring variant viruses were used to map the binding sites of the three MAbs. The three MAbs bound to overlapping but distinct epitopes on EDIII. Our empirical studies clearly demonstrate that the antibody binding and neutralization capacity of two MAbs was strongly influenced by naturally occurring mutations in DENV3. Our data demonstrate that the lateral ridge “type specific” epitope is not conserved between strains of DENV3. This variability should be considered when designing and evaluating DENV vaccines, especially those targeting EDIII.

## Author Summary

Dengue viruses are mosquito-borne flaviviruses and the agents of dengue fever and dengue hemorrhagic fever. It has been widely assumed that antibodies that neutralize dengue bind to regions on the viral envelope (E) protein that are conserved within each serotype. However, few studies have explored how natural variation influences dengue-antibody interactions. Mouse antibodies that strongly neutralize dengue bind to a region on domain III of E protein. This region has been the focus of much recent work because it might be the target of protective human antibodies as well. We compared a large number of E protein sequences and discovered that the region on E protein domain III targeted by neutralizing antibodies was highly variable between strains of dengue serotype 3. Using a panel of antibodies, we experimentally demonstrate that natural strain variation in dengue serotype 3 has a strong influence on antibody binding and neutralization. Our results challenge the dogma that neutralizing antibody binding regions are conserved within each serotype. The results of this study are relevant to the current global effort to develop and evaluate dengue vaccines.

## Introduction

Dengue viruses (DENVs) are mosquito-borne flaviviruses and the agents of dengue fever and dengue hemorrhagic fever (DHF). According to the World Health Organization, over 2.5 billion people are at risk of contracting dengue, 100 million people develop symptomatic infections and up to 50,000 die from DHF each year. The DENV complex consists of 4 serotypes (DENV1-DENV4). DENVs have antibody epitopes that are unique to each serotype and epitopes that are cross reactive between serotypes. People who have recovered from primary DENV infections develop long term, protective immune responses against the homologous serotype only. In fact, individuals exposed to a second infection with a different serotype face a greater risk of developing DHF indicating that pre-existing immunity can exacerbate disease under some conditions [1].

As previously infected individuals do not appear to be re-infected with the same serotype, it is widely assumed that neutralizing antibody epitopes are conserved among strains belonging to the same serotype [2],[3]. In fact, the current strategy for developing dengue vaccines is based on the assumption that a neutralizing immune response directed to a single strain will protect against most if not all strains of DENV within the serotype. However, there is considerable genetic diversity within each serotype such that each has been subdivided into genotypes [4]. Despite this diversity, surprisingly few studies have explored how naturally occurring strain variation within each serotype influences DENV neutralization. Blaney and colleagues immunized monkeys with candidate live attenuated dengue vaccines and characterized the immune response in monkeys by using a panel of viruses representing the 4 serotypes and genotypes within each serotype. They observed large differences in neutralization titer when comparing different genotypes of DENV3 [5]. In a study of pediatric dengue cases in Thailand, investigators observed significant differences in the ability of sera to neutralize reference and clinical strains of DENV3 [6]. Guzman and colleagues reported that amino acid sequence differences between DENV3 strains can have strong influences on virus neutralization by murine and human immune sera [7]. Studies with other flaviviruses have also demonstrated that neutralization is dependent on the lineages and strains used in the assay [8],[9]. Thus, the current paradigm that neutralizing antibody epitopes are conserved within each serotype may not accurately depict the complexity of the antigenic relationships, especially in DENV3.

Antibodies, in particular, have emerged as key effector molecules responsible for protective and pathogenic immune responses to DENV [1]. The DENV envelope (E) protein is the major target of neutralizing antibody [10]. E protein mediates attachment to host cells and low pH fusion of the viral and host cell membranes. The crystal structures of E from several flaviviruses (tick borne encephalitis, DENV2, DENV3 and West Nile) have been solved [11]–[14]. Individual subunits of E protein consist of three beta-barrel domains designated domains I (EDI), II (EDII) and III (EDIII) and the native protein is a homodimer [11],[12],[14]. Mouse monoclonal antibodies (MAbs) that bind to all three domains of DENV E have been generated and characterized [10], [15]–[17]. The most potent neutralizing MAbs bind to an epitope on the lateral ridge of EDIII of flaviviruses [10],[18],[19]. This epitope, which is not conserved between dengue serotypes, has been the focus of much recent work because it might be the target of the natural human immune response that leads to type specific neutralization. Investigators are also testing EDIII as a vaccine for inducing antibodies that neutralize a specific serotype, without inducing serotype cross reactive antibodies with potential for disease enhancement [20],[21].

In the present study, we have examined the phylogenetic relatedness of the E protein sequences from a large number of viruses representing the different genotypes of DENV3. Many surface exposed amino acids were variable between established genotypes of DENV3. Especially noteworthy was the observation that the EDIII lateral ridge, which is a known site targeted by neutralizing MAbs in related flaviviruses, was variable between DENV3 genotypes. We experimentally demonstrate that naturally occurring amino acid differences in DENV3 EDIII lead to differential binding and neutralization by MAbs.

## Materials and Methods

### Cells and viruses

*Aedes albopictus* C6/36 cells were maintained at 28C in MEM (Gibco) supplemented with 10% fetal bovine serum (FBS) (Gibco), penicillin (100 U/ml) and streptomycin (100 µg/ml) in the presence of 5% CO_2_. Human leukemic monocyte lymphoma cell line U937 expressing DC-SIGN (U937 DC-SIGN) were maintained at 37C in RPMI (Gibco) supplemented with 10% FBS, 50 mM beta mercaptoethanol, penicillin (100 U/ml) and streptomycin (100 µg/ml) in the presence of 5% CO_2_. All media were also supplemented with 0.1 mM non-essential amino acids (Gibco) and 2 mM glutamine (Gibco).

Working virus stocks were obtained by inoculating C6/36 mosquito cells in MEM (Gibco) supplemented with 2% FBS (Gibco), penicillin (100 U/ml) (Gibco) and streptomycin (100 µg/ml) (Gibco) 0.1 mM non-essential amino acids (Gibco) and 2 mM glutamine (Gibco) and growing the virus for eight days at 28C under 5% CO_2_. Supernatants were harvested, clarified by centrifugation and, supplemented with 15% FBS and stored in aliquots at −80C. Viral titers were determined by plaque assay on Vero-81 cells as previously described [22] and only stocks with a titer above 10^5^ PFU/ml were used in experiments. The reference virus strains used in the study were strains West Pacific 74 (DENV 1), S16803 (DENV2), CH53489 (DENV3) and TVP-360 (DENV4) routinely used in the DENV neutralization test. These viruses were obtained from Robert Putnak (Walter Reed Army Institute of Research, MD) and they have been passaged >10 times in different mammalian (Vero, Diploid fetal rhesus lung and Primary African Green monkey kidney cells) and insect (*Aedes albopictus* C6/36 cells) cell lines. For studies on different genotypes of DENV3, we also used UNC3043 (strain 059.AP-2 from Philippines, 1984), UNC 3009 (D2863, Sri Lanka 1989), and UNC3066 (strain 1342 from Puerto Rico 1977). These viruses were obtained from Dr. Duane Gubler and Claire Wong at CDC, Fort Collins, CO. These viruses had been passaged 3 times in *Aedes albopictus* C6/36 cells prior to being used in these studies.

### Monoclonal antibodies

MAbs 8A1 (IgG1) and 14A4 (IgG1) against DENV3 were provided by Robert Putnak (Walter Reed Army Institute of Research, MD). MAb1H9 (IgM) was provided by John Aaskov (Queensland University of Technology, Australia) [23]. MAb 1A1-D2 was provided by John Roehrig, (DVBID, CDC, Fort Collins, CO). MAbs 8A5 (IgG1) and 12C1 (IgG1) were generated for this study by immunizing mice with purified DENV3 strain CH53489.

### Purification of DENV

Vero-81 cells were inoculated with UNC 3043 (DENV3 -genotype I), CH53489 (DENV3 - genotype II), UNC 3009 (DENV3 - genotype III), or UNC3066 (DENV3 -genotype IV) at an MOI of 0.1. The virus-containing media was harvested 5–7 days after infection and centrifuged to pellet cell debris. The clarified media was laid on top of a 20% sucrose (wt/vol) cushion and centrifuged (72,000×*g* for 5 h) to pellet the virus. The virus pellet was allowed to dissolve overnight in PBS before layering on a 10%–40% iodixanol gradient and being centrifuged at 163,700×*g* for 120 min. The virus-containing fractions were harvested. PBS was added to the virus to dilute the iodixanol. The diluted solution was centrifuged (72,000×*g* for 5 h) to pellet the virus and remove the iodixanol. The virus pellet was resuspended in PBS and virus protein content was estimated by spectrophotometry. The virus was stored at −80°C.

### Expression and purification of recombinant EDIII (rEDIII)

Recombinant EDIII constructs were created using cDNA from the following virus strains to represent each serotype of DENV and genotypes of DENV3: West Pacific 74 (DENV 1), S16803 (DENV2), UNC 3043 (DENV3 -genotype I), CH53489 (DENV3 - genotype II), UNC 3009 (DENV3 - genotype III), UNC3066 (DENV3 -genotype IV), and TVP-360 (DENV4). Envelope gene fragments encoding EDIII from DENV1, DENV3, and DENV 4 (AA295–398) and DENV2 (AA297–399) were amplified using Vent polymerase (NEB, Ipswich, MA). Reverse primers used in the study were designed to introduce either Hind III (for DENV2–4) or PstI (for DENV1) restriction site at the 3′ ends of the PCR products. PCR products were digested with HindIII or PstI and cloned into pMAL c2X vector (NEB) to generate recombinant EDIII that is fused to maltose binding protein (MBP-EDIII) at the N terminus according to the manufacturer's instructions. MBP-EDIII were expressed in *E.coli* DH5α (Invitrogen) and purified using amylose resin affinity chromatography (NEB) according to the manufacturer's instructions.

### Site directed mutagenesis of rEDIII

Selected amino acids residues on rEDIII were mutated by site directed mutagenesis using Quickchange multi kit (Stratagene, La Jolla, CA). When selecting sites to mutate, we gave precedence to positions on loops and (not beta sheets) because we did not want to disrupt the overall folding of EDIII. Thus, of the 20 positions we mutated, 16 (301–303- N-terminal linker loop; 323, 325–330- BC loop; 357, 358, 361- DE loop; 380, 382, 383- FG loop) are located on loops that form the lateral ridge neutralizing epitope recognized by type specific neutralizing MAbs [15],[17]. We also mutated amino acids on the A strand (positions 304, 308, 310) because this strand forms a dengue subcomplex epitope recognized by neutralizing MAbs [15]. We mutated position 386 on the G strand because Serafin and Aaskov reported that mutations at this position lead to escape from 1H9 antibody used in the current study [23]. PCR primers were designed using QuikChange® Primer Design Program (www.stratagene.com) and PCR was conducted according to manufacturer's instruction. Single stranded pMal c2X plasmids (NEB, Ipswich, MA) encoding MBP-EDIII fusion proteins with amino acid substitutions were cloned into DH5α cells for expression and purification of mutant rEDIIIs. Substitution of amino acids in all mutant constructs was confirmed by sequencing. Expression and purification of mutant rEDIII proteins were essentially same as mentioned in the earlier section.

### Binding ELISA with recombinant EDIII or purified DENV antigens

ELISA plates were coated by adding 200 ng/well of purified EDIII-MBP protein or 75 ng/well of purified DENV antigen in Carbonate buffer (pH 9.0) and incubating the plates overnight at 4C. Rabbit anti MBP sera (New England Biolabs) was used to quantify binding of MBP-EDIII to plates. The flavivirus cross reactive MAb 4G2 was used to quantify binding of virus to plates. Two hundred nanograms of EDIII-MBP saturated binding to the ELISA plate and we did not observe appreciable differences in binding between different EDIII proteins created for this study. Similarly, 75 ng saturated virus binding to the plate and we did not observe appreciable differences in binding between different viruses used in the current study.

The plates were washed with Tris buffered saline with 0.2% Tween 20 (TBST wash buffer) and blocked with 3% normal goat serum (NGS) in Tris buffered saline with 0.05% Tween 20 (TBST blocking buffer) for 1 hour at 37C. Serially diluted MAbs in TBST blocking buffer were then added to each well and incubated for one hour at 37C. After washing 3 times with TBST wash buffer, the plates were incubated for one hour at 37C with alkaline phosphatase conjugated goat anti-mouse antibody (Sigma). Plates were washed 3 times with TBST wash buffer and developed by adding p-nitrophenyl phosphate substrate (Sigma). Optical density (OD) was measured at 405 nm using a spectrophotometer.

### Neutralization assays

The flow cytometry based neutralization protocol as described by Kraus, *et. al*., was used with modifications to determine 50% neutralization values for each antibody [22]. Same amount of virus (2×10^7^ genome equivalent copies) from each DENV3 genotype was used to infect cells in experiments comparing the neutralization activity of MAbs among the DENV genotypes. Different concentrations of MAbs were mixed with each virus strain in a 96 well tissue culture plate and incubated for one hour at 37C in the presence of 5% CO_2_. U937DC-SIGN cells (5×10^4^ cells in 100 µl) were introduced to each well and incubated for an additional 2 hours at 37C to allow virus to bind to cells. The cells were then washed with media and 200 µl of fresh media was added to each well and incubated for 24–72 hr at 37C with 5% CO_2_. After washing 2 times with PBS, the cells were fixed and permeabilized using CytoFix/Cytoperm kit (BD bioscience). The cells were then stained with Alexa 488 conjugated anti dengue MAb 2H2 and the percentage of infected cells was measured in a flow cytometer. EC50 values were calculated using GraphPad Prism version 4.00 for Windows (GraphPad Software, San Diego California USA, www.graphpad.com) and non linear regression analysis.

### DENV3 sequence analysis

**Figure 1:**
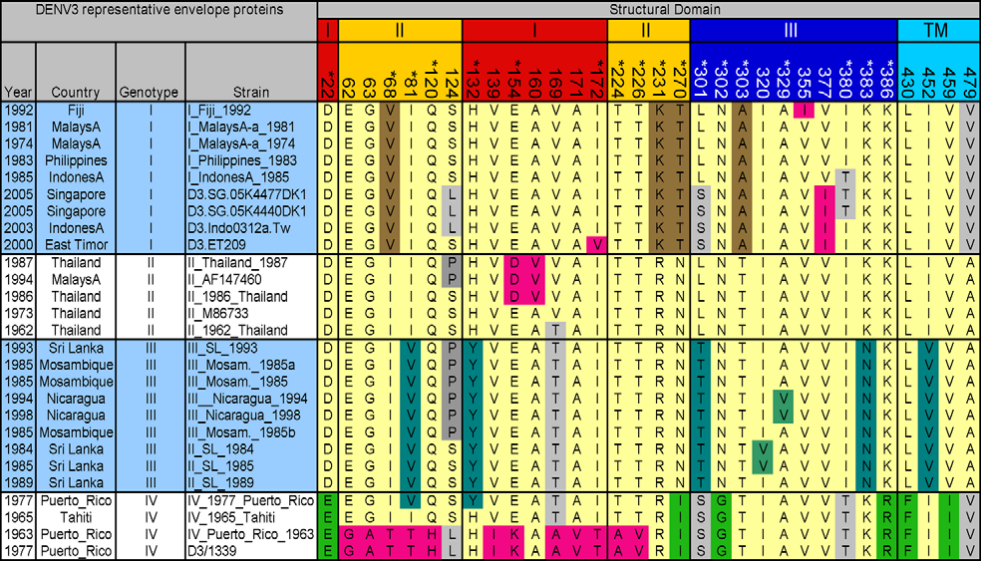
Informative sites in the envelope protein of dengue serotype 3. One hundred and seventy five DENV3 envelope protein sequences were aligned and 32 informative/variable sites were identified. The figure displays the informative sites and the variability within and between the different genotypes (I, II, III and IV) of DENV3 [26]. The envelope protein is divided into four domains indicated by the coloring on the position numbers. Red, domain I; yellow, domain II; blue, domain III; and cyan, transmembrane domain (TM). Residues that are unique to a given genotype are indicated by unique colors. Brown, genotype I; teal, genotype III; green, genotype IV; pink, unique polymorphisms; light yellow, predominant residues shared among multiple genotypes; and gray, variation shared among multiple genotypes.

A total of 175 unique DENV3 full-length envelope protein sequences were downloaded from GenBank and these were aligned using ClustalX version 1.83 [24] using the PAM distance matrix and default parameters. A variety of parameters and substitution matrices for the alignment were evaluated using the program TuneClustalv1.0 (http://www.homepage.mac.com/barryghall/Software.html) and the PAM series matrix was determined to be the most appropriate, with default gap opening and extension values. The alignment was used to identify 32 variable/informative sites that were defined as columns of heterogeneity in the alignment where the same amino acid change occurred in at least three independent sequences. To display the 32 informative sites (Figure 1), we selected a representative subset of 28 sequences that contained at least 4 sequences from each genotype of DENV3 depicted in Figure S1.

## Results

### Variable amino acids on DENV3 E protein

**Figure 2:**
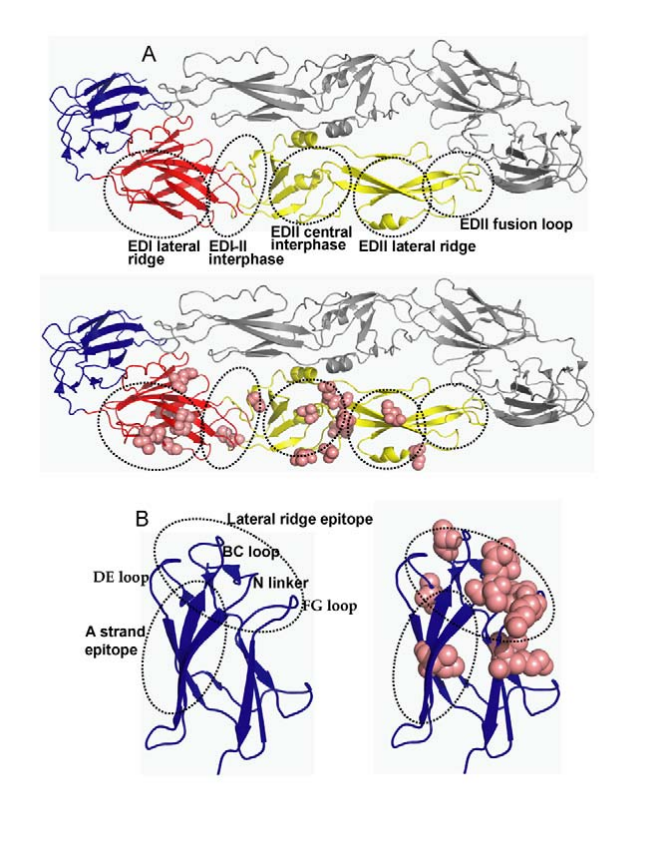
Location of MAb epitopes and informative sites on DENV3 E protein. The figure is based on the structure of the ectodomain of DENV3 E protein solved by Modis and colleagues. **A.** The flavivirus E protein consist of three beta-barrel domains designated domains I (red), II (yellow) and III (blue). The native protein is a homodimer that lies flat on the surface of the virus. The top image depicts the major antigenic sites on domains I and II (see Table 1 for details). The bottom image displays the location of informative sites on domains I and II (pink). **B.** An enlarged view of domain III displaying antigenic sites and informative sites. The left image displays the lateral ridge and A strand epitopes. The right image displays the domain III informative sites (pink).

As individuals infected with DENV appear to develop a long term, protective immune response to the homologous serotype, it has been assumed that neutralizing antibody epitopes are conserved within each serotype [10],[25]. To further evaluate this assumption, we used phylogenetic approaches to compare the full length E protein sequence of 175 DENV3 strains. The sequences, which were obtained from Genbank, had representatives of each of the 4 recognized genotypes of DENV3 [26]. Amino acid positions that were variable in 3 or more independent sequences as identified by alignment were defined as informative sites. Thirty two of the 493 amino acids on DENV3 E protein were identified to be informative sites (Figure 1). Individual subunits of E protein consist of three beta-barrel domains designated domains I (EDI), II (EDII) and III (EDIII) (Figure 2A). Informative sites were present in all domains of the E protein (Figures 1 and 2). Many of the informative sites had mutations that were conserved within but not between DENV3 genotypes (Figure 1). Twenty eight of the thirty two informative sites were located on the ectodomain (AA 1 to 392) of E protein. As the structure of the ectodomain of DENV3 E protein has been solved, we were able to determine if the variant sites were either surface exposed or buried [12]. Eighteen of 28 informative sites in the ectodomain were surface exposed, while others were partially or completely buried in the molecule (Figures 1 and 2).

### Relationship of DENV3 E protein informative sites to known antibody epitopes

We compared the locations of known antibody epitopes on the flavivirus E protein and the positions of informative sites on DENV3 E protein. Mouse monoclonal antibodies (MAbs) that bind to E protein have been mapped to six regions (Figure 2A and Table 1) [10],[18]. Most of the informative sites on EDI and EDII were within or adjacent to these antigenic regions (Figure 2A and Table 1). However, the antigenic region at the fusion loop was completely conserved between DENV3 strains (Table 1).

**Table 1:** Location of antigenic sites and informative sites on dengue type 3 E protein.

Domain of DENV3 E protein^1^	Antigenic region^2^	Flavivirus E protein mutations that influence mouse Mab binding^3^	DENV3 E protein informative sites within antigenic region^4^
EDI (AA 1–52,132–191,278–294)	Lateral Ridge	166–169, 179, 291	160, 169, 171, 172
	EDI-EDII interphase	49–52, 136, 184–187,268–277	132, 139, 270
EDII (AA 53–131, 192–278)	Central Region	123–128, 210, 215, 232–233	62, 62, 120, 124,224, 226, 231
	Lateral Ridge	67–72, 75–76, 81–83, 86, 112	68, 81
	Fusion Loop	99–107	
EDIII (AA 295–392)	Lateral Ridge	301–303, 327–330, 381–382	301–303, 329, 355, 380, 383
	A strand	305–308	

1 The amino acids that form each domain of DENV3 E protein are according to Modis et al [12].

2 The antigenic regions are based on the assignments and nomenclature used by Pierson et al [19].

3 Based on Roehrig and Pierson [10],[19].The numbering is based on the DENV3 E protein sequence.

4 DENV3 E protein informative sites were identified as described in Figure 1.

Many MAbs that strongly neutralize flaviviruses bind to EDIII. DENV serotype specific (type specific), neutralizing MAbs bind to epitopes on the lateral ridge of EDIII, which is formed by three loops connecting the D–E, B–C, F–G beta sheets on EDIII and the linker region connecting EDIII and EDI (Figure 2B and Table 1) [10],[18]. Investigators have also defined an epitope on EDIII recognized by MAbs that neutralize more than one DENV serotype [15],[27],[28]. This DENV sub complex epitope overlaps with the lateral ridge epitope but is centered at positions 305–308 (DENV3 numbering) on the A strand of EDIII (Figure 2B and Table 1). We compared the positions of known antibody epitopes and DENV3 informative sites on EDIII. The dengue type specific, lateral ridge epitope overlapped extensively with the informative sites on EDIII (Figure 2B, Table 1). This analysis supports the hypothesis that the EDIII lateral ridge epitope engaged by strongly neutralizing MAbs is not conserved between DENV3 strains.

### Mapping of DENV3, EDIII reactive antibodies

**Figure 3:**
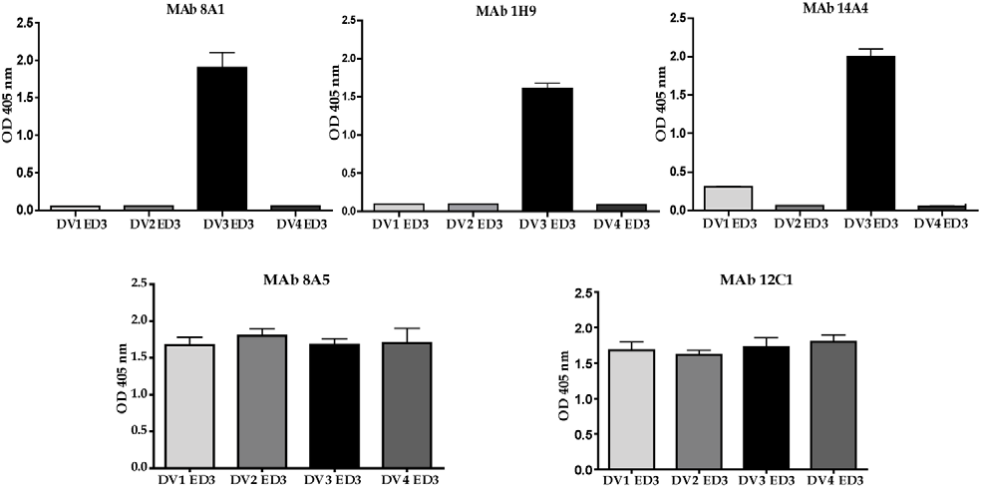
Binding of mouse MAbs to recombinant EDIII from the 4 serotypes of DENV. MAb binding was detected by ELISA. MAbs 8A1 and 1H9 bound to EDIII from DENV3 only. MAb 14A4 bound to EDIII from DENV3 and to a lesser extent to EDIIII from DENV1. MAbs 8A5 and 12C1 bound to EDIII from all 4 serotypes.

To directly address if natural amino acid variation in DENV3 EDIII results in altered antibody binding and neutralization, we assembled and mapped a panel of DENV3 EDIII reactive mouse MAbs. All the antibodies selected for this study (8A1, 1H9, 14A4, 8A5, and 12C1) bound to EDIII of DENV3 (Figure 3). MAbs 8A1 and 1H9 were type specific as they only bound to EDIII from DENV3 (Figure 3) and only neutralized DENV3 (data not shown). 14A4 was a sub-complex specific antibody that bound strongly to DENV3 and weakly to DENV1 (Figure 3). 14A4 strongly neutralized DENV3 and weakly neutralized DENV1 (data not shown). 8A5 and 12C1 were non-neutralizing antibodies that cross reacted with EDIII from all 4 serotypes (Figure 3 and data not shown).

**Figure 4:**
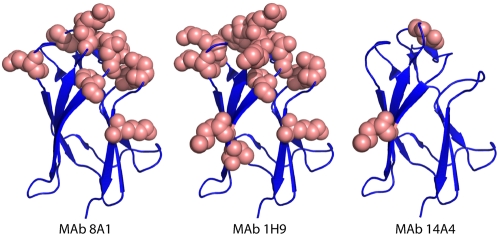
Mapping EDIII epitopes for MAbs 8A1, 1H9 and 14A4. The Figure depicts the positions of mutations that reduced MAb binding by >80%. Many mutations, mainly on the lateral ridge, reduced binding of 8A1 and 1H9. In contrast only three mutations inhibited binding of 14A4.

To map the binding sites of the MAbs, we expressed and purified 28 EDIII recombinant proteins with defined mutations. The positions to mutate were selected based on antibody mapping studies done with other flaviviruses [10],[15],[17],[27],[29],[30]. The binding of each antibody to wild type and mutant proteins was compared by ELISA (Table 2). MAb 1H9 is a type specific, neutralizing DENV3, EDIII reactive IgM antibody that has previously been shown to select for escape mutation at position 386 [23]. We observed a greater than 80% loss of binding of 1H9 when amino acids at positions 302, 304, 308, 310, 323, 325–330, 357, 358, 361, 380, 382 and 386 were mutated on EDIII (Table 2). Most of these mutations are on the lateral ridge of EDIII (Figure 4).

**Table 2:** Binding^1^ of mouse MAbs to mutant DENV3 EDIII proteins.

Mutation	Mouse MAb				
	1H9	8A1	14A4	8A5	12C1
I301A	97	**13** 2	100	100	99
I301G	52	**3**	97	100	100
N302A	**9**	**4**	95	100	99
N302G	223	**12**	100	98	99
T303A	107	77	100	100	100
T303G	43	42	100	100	98
F304A	**15**	**5**	30	90	100
K308A	**16**	37	**5**	100	96
V310A	**16**	54	83	97	100
E323G	**17**	69	99	100	95
K325A	57	74	38	99	98
K325G	**12**	25	80	90	100
G326A	**10**	**5**	**18**	96	97
E327A	**13**	**4**	72	98	100
D328A	**10**	**4**	**5**	93	100
A329G	**11**	28	74	100	96
P330A	**11**	**6**	29	95	100
T357A	35	76	100	97	98
T357G	**13**	47	100	99	97
K358G	**17**	50	100	95	100
E361G	**6**	**10**	100	98	100
I380A	**18**	**5**	97	98	96
I380G	**4**	**4**	88	96	98
D382G	**5**	**15**	90	98	95
K383A	106	92	100	100	99
K383G	101	97	99	99	100
K386A	**4**	35	97	96	98
K386G	**4**	**15**	96	98	100

1 Binding to each mutant expressed as a percentage of binding to wild type EDIII protein from genotype II.

2 The values in bold indicate mutations that reduced MAb biding by >80% compared to the wild type EDIII.

MAb 8A1 is a strongly neutralizing, type specific DENV3, EDIII reactive IgG antibody. With this antibody, we observed a greater than 80% loss of binding when amino acids at 301, 302, 304, 326–328, 330, 361, 380, 382 and 386 were mutated on EDIII (Table 2). As in the case of 1H9, most of these positions overlap with the EDIII lateral ridge epitope. However, 1H9 and 8A1 did not bind to identical epitopes because some mutations that influenced 1H9 had marginal to no effect on 8A1 (Table 2).

MAb 14A4 is a neutralizing EDIII reactive IgG antibody that cross-reacts with DENV3 and DENV1 (Figure 3). This DENV sub complex antibody bound poorly to recombinant proteins with mutations at position 308 (A strand), and positions 326 and 328 (B–C loop) (Table 2). These mutations are located at a similar position to a DENV EDIII sub complex epitope recently described in the literature [15],[27]. The sub complex epitope overlaps with the lateral ridge but is centered on the A strand of EDIII.

The DENV complex cross reactive MAbs (8A5, 12C1) bound to all the mutant proteins indicating these antibodies likely bind to a cross reactive epitope outside the lateral ridge region (Table 2). In Figure 4 we display the structure of DENV3 EDIII and the location of mutations that reduced the binding (>80%) of each MAb.

One concern with the above mapping studies was that some mutations might disrupt the overall folding of EDIII and non-specifically reduce antibody binding. To address this concern, we performed binding studies with a well characterized DENV subcomplex specific MAb 1A1D2, which binds to and neutralizes DENV1, 2 and 3 but not 4 [15]. The crystal structure of DENV2-EDIII-1A1-D2 Fab complex has been solved [31]. The 1A1-D2 MAb binds to a highly conformational epitope with a footprint that consists of the A strand, B strand, DE loop and G strand of EDIII [31]. When we compared the binding of 1A1-D2 to the panel of DENV3 EDIII mutants created for this study, a greater than 80% loss of binding was observed when DENV3 EDIII positions 304, 308, 310, 326, 328 and 330 were mutated (Table S1). DENV3 positions 304–310 are on the A strand and positions 326, 328 and 330 are on the BC loop which is adjacent to the B strand. The EDIII mutations at a distance from the known footprint of 1A1-D2 did not disrupt the highly conformational 1A1-D2 epitope indicating that the overall folding of EDIII was preserved in our mutants (Table S1).

### Binding of MAbs to different genotypes of DENV3

**Figure 5:**
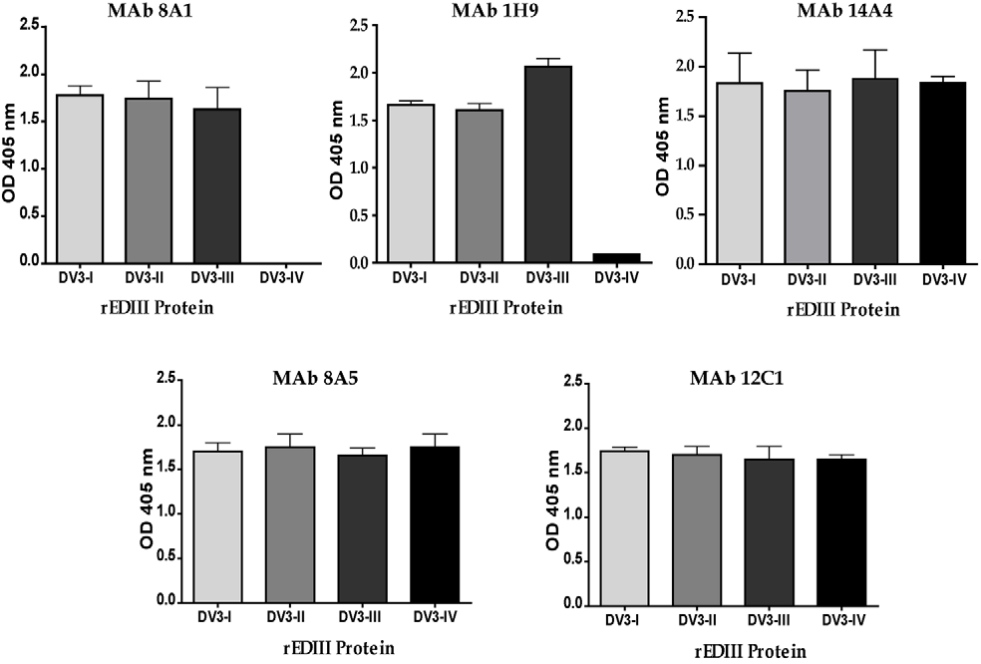
Binding of mouse MAbs to recombinant EDIII from the 4 genotypes of DENV3. MAb binding was detected by ELISA. MAbs 8A1 and 1H9 bound to EDIII from DENV3 genotypes I, II and III but not to IV. MAbs 14A4, 8A5 and 12C1 bound to all 4 genotypes.

**Figure 6:**
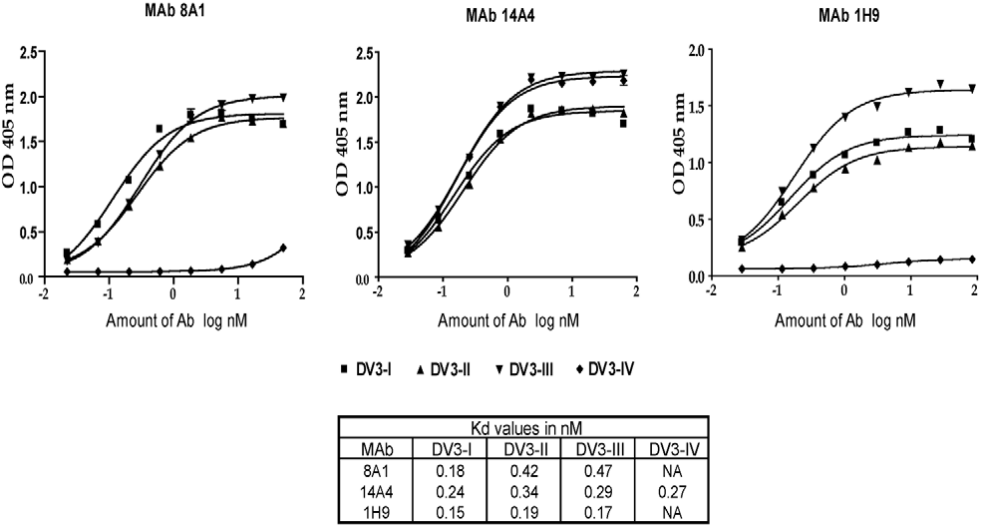
Binding of mouse MAbs to DENV3 genotypes. DENV3 genotype I (DV3-I), genotype II (DV3-II), genotype III (DV3-III) and genotype IV (DV3-IV) viruses were purified and used in binding assays with MAb 8A1, 14A4 and 1H9. MAb 14A4 bound to all 4 genotypes with similar apparent affinity. MAbs 8A1 and 1H9 bound to DENV3, genotypes I, II and III with similar apparent affinity, while no binding was detected with genotype IV virus.

Several amino acid positions (301, 302, 329, 380 and 386) on EDIII implicated in binding to MAbs 8A1 and 1H9 (Table 2) were also identified as informative sites that were not conserved between DENV3 genotypes (Figure 1). All these positions are located in close proximity to one another on the EDIII lateral ridge. To directly address if naturally occurring variation at these informative sites leads to altered antibody interactions, we compared the binding of MAbs 1H9, 8A1 and 14A4 to representative EDIII from each of the 4 genotypes of DENV3. MAbs 8A1 and 1H9 bound to genotypes I, II and III but not to EDIII from genotype IV (Figure 5). The DENV sub complex specific 14A4 antibody bound to EDIII from all 4 genotypes (Figure 5). Studies were also performed to compare the binding of these MAbs to purified viruses (Figure 6). As predicted from the recombinant EDIII binding experiments, 1H9 bound to DENV3 genotype I, II and III viruses but not to the genotype IV virus (Figure 6). Similarly, MAb 8A1 bound to genotypes I, II, and III but not to the genotype IV (Figure 6). MAb 14A4 bound to all 4 genotypes. These results indicate that naturally occurring amino acid variation on DENV3 EDIII influence the binding of type specific antibodies.

**Figure 7:**
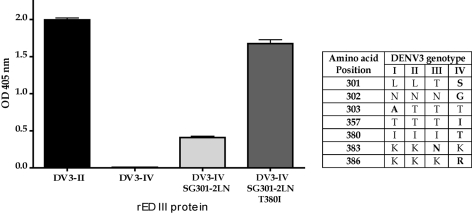
Identification of naturally occurring mutations that reduce binding of MAb 8A1. The table displays the EDIII amino acid differences between the different genotypes of DENV3. MAb 8A1 bound to EDIII from DENV3 genotype II (DV3-II) but not genotype IV (DV3-IV). Binding was partially restored when positions 301 and 302 were changed from the genotype IV to genotype II (DV3-IV SG301-2LN)). Complete binding was restored when positions 301, 302 and 380 were changed (DV3-IV SG301-2LN, T380I).

To verify that amino acid differences at the EDIII lateral ridge were responsible for MAb binding differences, further studies were conducted with MAb 8A1 and recombinant EDIII proteins. We systematically changed amino acids in the EDIII genotype IV construct to genotype II and defined the minimum number of changes required to restore the 8A1 epitope. In Figure 7 we depict the EDIII amino acid differences between the different genotypes. Simply making single amino acids changes at positions 301 or 302 did not restore binding. Some binding was regained when both 301 and 302 were changed from SG (genotype IV) to LN (genotype II) (Figure 7). Full binding was restored when positions 301, 302 and 380 were changed (Figure 7) indicating that these were the critical changes that led to the loss of binding of MAb 8A1 to DENV3 genotype IV. Residues 301, 302 and 380 are surface-exposed neighbors on the lateral ridge of EDIII, with residues 301 and 380 separated by approximately 4.7 angstroms. Thus, these three residues are likely to be a part of a single epitope bound by 8A1.

### Neutralization of DENV3 genotypes by MAbs

Experiments were conducted to compare the ability of EDIII MAbs to neutralize different genotypes of DENV3. MAbs 8A1 and 1H9 failed to neutralize DENV3 genotype IV (Table 3), which was expected since these antibodies did not bind to this virus (Figure 6). Surprisingly, even though we did not observe differences in the binding of 8A1 and 1H9 to genotype I, II and III viruses, we observed differences in the neutralization titers (Table 3). For example the neutralization titers for 8A1 were 10 fold different between genotype I and III viruses (Table 3). 1H9 displayed a 60 fold difference in the neutralization titer between genotype I and II viruses (Table 3). These results indicate that two mechanisms influence the ability of MAbs to neutralize virus infectivity. In the first, mutations which ablate binding also ablate neutralization. In the second, genetic differences between DENV3 strains that have little effect on in vitro binding can have significant biological effects on neutralization.

**Table 3:** Neutralization of DENV3 genotypes by EDIII MAbs.

MAb	50% Neutralization titer^1^ (µg/ml ± SEM)			
	DENV3 genotype			
	I	II	III	IV
8A1	0.43±0.15	1.3±0.32	4.4±0.67	NN^2^
1H9	0.02±0.002	1.2±0.69	0.37±0.02	NN
14A4	0.64±0.34	1.8±0.54	2.2±0.18	0.25±0.02

1 50% neutralization titer determined using U937+DC-SIGN expressing cells.

2 Not neutralized.

## Discussion

A long-held paradigm in flavivirus research has been that DENVs display little if any within intra-serotypic antigenic variation and this has been the basis for the development of current multivalent vaccines and immunotherapeutics [10],[18]. The main goal of the current study was to characterize the extent of envelope protein variation within the DENV3 serotype and to determine if this variation influenced antibody binding and neutralization. Sequence and structural analysis of the E protein indicated that 7% of the amino acids were variable between the four genotypes of DENV3, and most of the non-conserved residues were surface exposed and located at or proximal to known antibody binding sites. Particularly striking was the variation observed on the lateral ridge of domain III, which has previously been identified as the target of antibodies that strongly neutralize flaviviruses [10],[18]. Finally, we demonstrated that natural variation on EDIII influences the ability of MAbs to bind and neutralize DENV3. Our results reported here, together with the other published studies [8],[9] challenge the long held view that neutralizing antibody epitopes are conserved across DENV strains belonging to the same serotype.

Our results show that EDIII lateral ridge antibodies 8A1 and 1H9 bound to DENV3 genotypes I, II and III but not genotype IV indicating that naturally occurring mutations in EDIII can lead to a total loss of MAb binding. Even though MAbs 8A1 and 1H9 bound to DENV3 genotypes I, II and III with similar apparent affinity, we observed striking differences in the ability of the MAbs to neutralize these viruses. The neutralization titers were almost 10 fold different between viruses for 8A1 and 60 fold different for 1H9. Our results indicate that apparent affinity of antibody to virus immobilized on ELISA wells is not always predictive of the neutralization titer. There are amino acid differences on the EDIII lateral ridge of genotype I, II and III viruses (Figure 7) and these changes may lead to subtle changes in virus antibody interactions that are not detected in our ELISA binding assay. The flavivirus envelope proteins undergo low pH induced conformational changes during viral entry [32]. Some antibodies neutralize flaviviruses by binding to the virus in endosomes and blocking late steps in viral entry [29]. It is possible that antibody binding to the low pH conformation of the viral envelope might be a better predictor of neutralization potency than binding to the neutral pH conformation assessed here. Further studies with virions in different conformations, and an infectious clone of DENV3 to introduce targeted mutations are needed to dissect the mechanism underpinning the ability of EDIII lateral ridge antibodies to neutralize different genotypes of DENV3.

One potential problem with our studies is the possibility that some of the recombinant EDIII proteins used in the current study might be grossly misfolded and the binding differences might not be due to direct interactions between antibody and the altered amino acid. NMR and antibody binding assays have established that wild type EDIII expressed as an MBP fusion protein is properly folded [33]–[36]. When selecting sites to mutate, we primarily targeted surface exposed amino acids on loops because we did not want to disrupt the overall structure of EDIII. Moreover, in most cases mutations that led to the loss of binding of 8A1 or 1H9 preserved the binding sites of 14A4, 8A5 and 12C1 indicating that the proteins were not grossly misfolded (Table 2). Finally we probed the conformation of our EDIII mutants using a DENV sub complex antibody 1A1-D2 which binds to a highly conformational epitope on EDIII that has been mapped by X-ray crystallography [31]. As depicted in Table S1, of the 28 mutant proteins we created only 6 mutants failed to bind to this antibody (>80% loss of binding). The 6 mutants that failed to bind had mutations that were on or adjacent to the known footprint of 1A1-D2. Based on these results we are confident that the recombinant proteins used in the current study were not grossly misfolded. Nevertheless, we cannot completely rule out indirect or distance effects of some mutations on antibody binding and some of the mutations that reduced binding might not be in direct contact with antibody.

Several groups have focused on developing DENV vaccines based on recombinant EDIII [21],[37],[38]. Our results indicate that EDIII based vaccines need to be carefully evaluated. If people immunized with these antigens mainly develop neutralizing antibodies that bind to the lateral ridge epitope recognized by MAbs such as 8A1 and 1H9, then natural strain variation is likely to lead to vaccine failure. Alternatively, if EDIII vaccines stimulate antibodies to a conserved, neutralizing epitope such as the A strand epitope (recognized by 14A4), then the vaccine might be broadly protective across DENV3 strains.

Recently we reported that people exposed to natural DENV infections have low levels of EDIII reactive antibody several years after recovery from infection [36]. Given the low levels of EDIII reactive antibody in human immune sera, we were surprised by the extent of amino acid variation between EDIII from different DENV3 genotypes. It is plausible that interactions with cellular receptors and not antibody are behind the observed variability in EDIII. It is also plausible that EDIII reactive, neutralizing antibodies are abundant during early stages after infection and select for mutation in EDIII. Further studies are needed to better characterize human receptors and antibodies that interact with E protein and to assess how these interactions contribute to natural variation in DENV3 E protein.

## Supporting Information

Figure S1 Phylogenetic tree of 175 DENV3 E protein sequences used to identify informative sites. A phylogenetic tree was generated using Bayesian inference to analyze the evolutionary relationship of 175 unique DENV3 envelope protein amino acid sequences that were available from Gen Bank at the time this study was initiated. The four known genotypes of DENV3 are indicated. The numeric values at the nodes represent Bayesian posterior probabilities and the distance scale bar represents 0.01 changes per site.
(0.15 MB PDF)

Table S1 Binding of mouse MAbs 1A1-D2 to mutant DENV3 EDIII proteins
(0.01 MB PDF)

## References

[1] Halstead SB (2007). Dengue.. Lancet.

[2] Sabin AB (1952). Research on dengue during World War II.. Am J Trop Med Hyg.

[3] Imrie A, Meeks J, Gurary A, Sukhbaatar M, Truong TT (2007). Antibody to Dengue 1 Detected More Than 60 Years after Infection.. Viral Immunology.

[4] Rico-Hesse R (2003). Microevolution and virulence of dengue viruses.. Adv Virus Res.

[5] Blaney JE, Matro JM, Murphy BR, Whitehead SS (2005). Recombinant, live-attenuated tetravalent dengue virus vaccine formulations induce a balanced, broad, and protective neutralizing antibody response against each of the four serotypes in rhesus monkeys.. J Virol.

[6] Endy TP, Nisalak A, Chunsuttitwat S, Vaughn DW, Green S (2004). Relationship of preexisting dengue virus (DV) neutralizing antibody levels to viremia and severity of disease in a prospective cohort study of DV infection in Thailand.. J Infect Dis.

[7] Zulueta A, Martin J, Hermida L, Alvarez M, Valdes I (2006). Amino acid changes in the recombinant Dengue 3 Envelope domain III determine its antigenicity and immunogenicity in mice.. Virus Res.

[8] Li L, Barrett AD, Beasley DW (2005). Differential expression of domain III neutralizing epitopes on the envelope proteins of West Nile virus strains.. Virology.

[9] Sanchez MD, Pierson TC, McAllister D, Hanna SL, Puffer BA (2005). Characterization of neutralizing antibodies to West Nile virus.. Virology.

[10] Roehrig JT (2003). Antigenic structure of flavivirus proteins.. Adv Virus Res.

[11] Modis Y, Ogata S, Clements D, Harrison SC (2003). A ligand-binding pocket in the dengue virus envelope glycoprotein.. Proc Natl Acad Sci U S A.

[12] Modis Y, Ogata S, Clements D, Harrison SC (2005). Variable surface epitopes in the crystal structure of dengue virus type 3 envelope glycoprotein.. J Virol.

[13] Nybakken GE, Nelson CA, Chen BR, Diamond MS, Fremont DH (2006). Crystal structure of the West Nile virus envelope glycoprotein.. J Virol.

[14] Rey FA, Heinz FX, Mandl C, Kunz C, Harrison SC (1995). The envelope glycoprotein from tick-borne encephalitis virus at 2 A resolution.. Nature.

[15] Sukupolvi-Petty S, Austin SK, Purtha WE, Oliphant T, Nybakken GE (2007). Type- and Subcomplex-Specific Neutralizing Antibodies against Domain III of Dengue Virus Type 2 Envelope Protein Recognize Adjacent Epitopes.. J Virol.

[16] Crill WD, Chang G-JJ (2004). Localization and Characterization of Flavivirus Envelope Glycoprotein Cross-Reactive Epitopes.. J Virol.

[17] Gromowski GD, Barrett ADT (2007). Characterization of an antigenic site that contains a dominant, type-specific neutralization determinant on the envelope protein domain III (ED3) of dengue 2 virus.. Virology.

[18] Pierson TC, Fremont DH, Kuhn RJ, Diamond MS (2008). Structural insights into the mechanisms of antibody-mediated neutralization of flavivirus infection: implications for vaccine development.. Cell Host Microbe.

[19] Pierson TC, Diamond MS (2008). Molecular mechanisms of antibody-mediated neutralisation of flavivirus infection.. Expert Rev Mol Med.

[20] Hermida L, Bernardo L, Martin J, Alvarez M, Prado I (2006). A recombinant fusion protein containing the domain III of the dengue-2 envelope protein is immunogenic and protective in nonhuman primates.. Vaccine.

[21] Etemad B, Batra G, Raut R, Dahiya S, Khanam S (2008). An Envelope Domain III-based Chimeric Antigen Produced in Pichia pastoris Elicits Neutralizing Antibodies Against All Four Dengue Virus Serotypes.. Am J Trop Med Hyg.

[22] Kraus AA, Messer W, Haymore LB, de Silva AM (2007). Comparison of Plaque- and Flow Cytometry- Based Methods for Measuring Dengue Virus Neutralization.. J Clin Microbiol.

[23] Serafin IL, Aaskov JG (2001). Identification of epitopes on the envelope (E) protein of dengue 2 and dengue 3 viruses using monoclonal antibodies.. Archives of Virology.

[24] Chenna R, Sugawara H, Koike T, Lopez R, Gibson TJ (2003). Multiple sequence alignment with the Clustal series of programs.. Nucl Acids Res.

[25] Halstead SB (2003). Neutralization and antibody-dependent enhancement of dengue viruses.. Adv Virus Res.

[26] Lanciotti RS, Lewis JG, Gubler DJ, Trent DW (1994). Molecular evolution and epidemiology of dengue-3 viruses.. J Gen Virol.

[27] Gromowski GD, Barrett ND, Barrett AD (2008). Characterization of dengue virus complex-specific neutralizing epitopes on envelope protein domain III of dengue 2 virus.. J Virol.

[28] Rajamanonmani R, Nkenfou C, Clancy P, Yau YH, Shochat SG (2009). On a mouse monoclonal antibody that neutralizes all four dengue virus serotypes.. J Gen Virol.

[29] Nybakken GE, Oliphant T, Johnson S, Burke S, Diamond MS (2005). Structural basis of West Nile virus neutralization by a therapeutic antibody.. Nature.

[30] Beasley DW, Barrett AD (2002). Identification of neutralizing epitopes within structural domain III of the West Nile virus envelope protein.. J Virol.

[31] Lok SM, Kostyuchenko V, Nybakken GE, Holdaway HA, Battisti AJ (2008). Binding of a neutralizing antibody to dengue virus alters the arrangement of surface glycoproteins.. Nat Struct Mol Biol.

[32] Mukhopadhyay S, Kuhn RJ, Rossmann MG (2005). A structural perspective of the flavivirus life cycle..

[33] Volk DE, Beasley DW, Kallick DA, Holbrook MR, Barrett AD (2004). Solution structure and antibody binding studies of the envelope protein domain III from the New York strain of West Nile virus.. J Biol Chem.

[34] Volk DE, Lee YC, Li X, Thiviyanathan V, Gromowski GD (2007). Solution structure of the envelope protein domain III of dengue-4 virus.. Virology.

[35] Yu S, Wuu A, Basu R, Holbrook MR, Barrett AD (2004). Solution structure and structural dynamics of envelope protein domain III of mosquito- and tick-borne flaviviruses.. Biochemistry.

[36] Wahala WM, Kraus AA, Haymore LB, Accavitti-Loper MA, de Silva AM (2009). Dengue virus neutralization by human immune sera: role of envelope protein domain III-reactive antibody.. Virology.

[37] Babu JP, Pattnaik P, Gupta N, Shrivastava A, Khan M (2008). Immunogenicity of a recombinant envelope domain III protein of dengue virus type-4 with various adjuvants in mice.. Vaccine.

[38] Bernardo L, Hermida L, Martin J, Alvarez M, Prado I (2008). Anamnestic antibody response after viral challenge in monkeys immunized with dengue 2 recombinant fusion proteins.. Arch Virol.

