# Supplementary figures and images for: Natural Strain Variation and Antibody Neutralization of Dengue Serotype 3 Viruses
Source: PLoS Pathog. 2010 Mar 19;6(3):e1000821. doi: 10.1371/journal.ppat.1000821 (PMC2841629; doi:10.1371/journal.ppat.1000821)

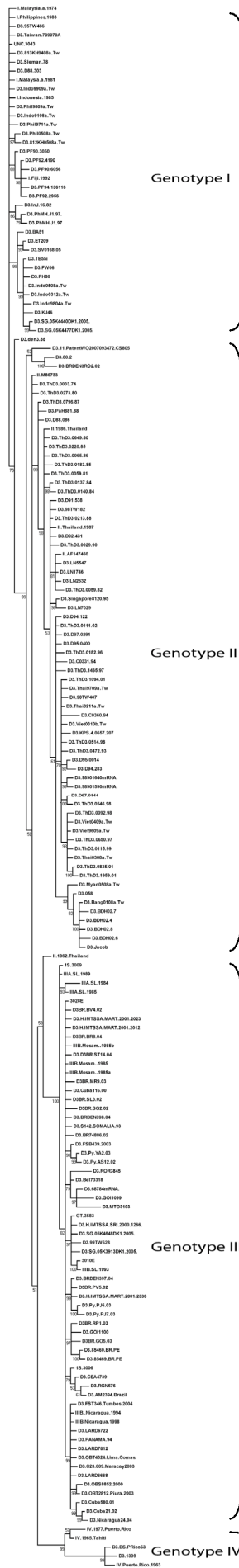

Supplement: Figure S1 — Phylogenetic tree of 175 DENV3 E protein sequences used to identify informative sites. A phylogenetic tree was generated using Bayesian inference to analyze the evolutionary relationship of 175 unique DENV3 envelope protein amino acid sequences that were available from Gen Bank at the time this study was initiated. The four known genotypes of DENV3 are indicated. The numeric values at the nodes represent Bayesian posterior probabilities and the distance scale bar represents 0.01 changes per site. (0.15 MB PDF) [file ppat.1000821.s001.pdf]
