# Supplementary material for: Natural Strain Variation and Antibody Neutralization of Dengue Serotype 3 Viruses
Source: PLoS Pathog. 2010 Mar 19;6(3):e1000821. doi: 10.1371/journal.ppat.1000821 (PMC2841629; doi:10.1371/journal.ppat.1000821)
Supplement: Table S1 — Binding of mouse MAbs 1A1-D2 to mutant DENV3 EDIII proteins (0.01 MB PDF) [file ppat.1000821.s002.pdf]

Table S1. Binding of mouse MAbs 1A1-D2 to mutant DENV3 EDIII proteins

Mutation	MAb 1A1-D2 Binding[1]
I301A	112.90
I301G	101.67
N302A	63.70
N302G	132.26
T303A	104.70
T303G	109.24
**F304A**[2]	**17.31**
**K308A**	**13.28**
E309A	78.55
V310A	21.71
E323G	118.07
K325A	32.61
K325G	26.14
**G326A**	**14.80**
E327A	63.22
**D328A**	**16.07**
A329G	45.19
**P330A**	**16.24**
T357A	92.80
T357G	77.53
K358G	86.87
E361G	49.49
I380G	99.17
D382G	64.42
K383A	85.78
K383G	86.01
K386A	60.02
K386G	47.75

[1] Binding to each mutant expressed as a percentage of binding to wild type EDIII protein from genotype II

[2] The values in bold indicate mutations that reduced MAb biding by >80% compared to the wild type EDIII
